# Observation of symptomatic thromboembolic events in endoscopic retrograde cholangiopancreatography patients with interruption of antithrombotic therapies

**DOI:** 10.3389/fmed.2025.1453026

**Published:** 2025-02-06

**Authors:** Jinqing Wu, Jianglong Hong, Hao Ding, Qiao Mei

**Affiliations:** ^1^Department of Gastroenterology, The First Affiliated Hospital of Anhui Medical University, Hefei, China; ^2^Department of Gastroenterology, Fuyang People’s Hospital, Fuyang, China

**Keywords:** endoscopic retrograde cholangiopancreatography, symptomatic thromboembolic events, bleeding, antithrombotic therapies, high-risk conditions

## Abstract

**Background and objectives:**

An increasing number of patients with antithrombotic therapies are undergoing endoscopic retrograde cholangiopancreatography (ERCP). Interruption of antithrombotic therapies may be associated with a higher risk of symptomatic thromboembolic (TE) events. We aimed to investigate the risk of symptomatic TE events among patients undergoing ERCP.

**Methods:**

A retrospective cohort study on patients at risk for symptomatic TE events who had undergone ERCP from January 2016 to October 2023 was conducted. A total of 2,482 patients who had undergone ERCP were included in this study. We compared the risk of symptomatic TE events within 30 days after ERCP between the group treated with antithrombotic agent and the group not treated with antithrombotic agent using multivariate regression analysis adjusted for covariates.

**Results:**

A total of 15 patients (0.60%, 15/2,482) developed symptomatic TE events within 30 days after ERCP. The symptomatic TE event rate in subjects on any antithrombotic drug was 1.46% with an odds ratio (OR) of 5.267 (*n* = 689, 95% CI 1.79–15.46, *p* = 0.002), compared with those not treated with antithrombotic drugs (*n* = 1,793). The symptomatic TE event rate in subjects on temporary interruption of antithrombotic drugs was 1.48% with an OR of 5.36 (*n* = 677, 95% CI 1.83–15.74, *p* = 0.002), compared with those not treated with antithrombotic drugs (*n* = 1,793). Multivariate regression analysis indicated that patients with high-risk conditions had a significantly higher risk of post-ERCP symptomatic TE events (adjusted OR 11.73, 95% CI 2.23–61.70).

**Conclusion:**

Interruption of antithrombotic drugs is associated with higher post-ERCP symptomatic TE events, particularly in high-risk conditions.

## Introduction

Endoscopic retrograde cholangiopancreatography (ERCP) is a common option for treating biliary and pancreatic duct diseases. The primary complications of ERCP include acute pancreatitis, bleeding, and other adverse events (1). The risk of bleeding (2–4) may be increased by using antithrombotic agents (4, 5), but the occurrence of thromboembolic (TE) events after ERCP is often fatal. Thus, the perioperative management of antithrombotic agents when conducting ERCP is a matter of great concern (6, 7). The current American Society for Gastrointestinal Endoscopy guidelines recommend the continuation of antithrombotic agents in low-risk endoscopic procedures and discontinuation of antithrombotic agents before high-risk endoscopic procedures (8, 9).

An increasing number of individuals who require ERCP are taking antithrombotic agents, and some cannot safely interrupt those agents due to the high risk of TE events. However, heparin bridging therapy increases the incidence of delayed bleeding (10, 11). It is unknown whether transient withdrawal of antithrombotic agents carries an unacceptable risk of TE events for patients undergoing ERCP. Yet, the long interruption of anticoagulants for more than 48 h is associated with TE events. There are conflicting data considering the risk of bleeding after ERCP in the presence of antithrombotic agents (12). Therefore, our study was carried out to determine the association between the interruption of antithrombotic therapies and symptomatic TE events in patients undergoing ERCP. We also sought to identify the potential risk of symptomatic TE events and search for novel methods for their prevention.

## Methods

### Study design and patients

**Figure 1:**
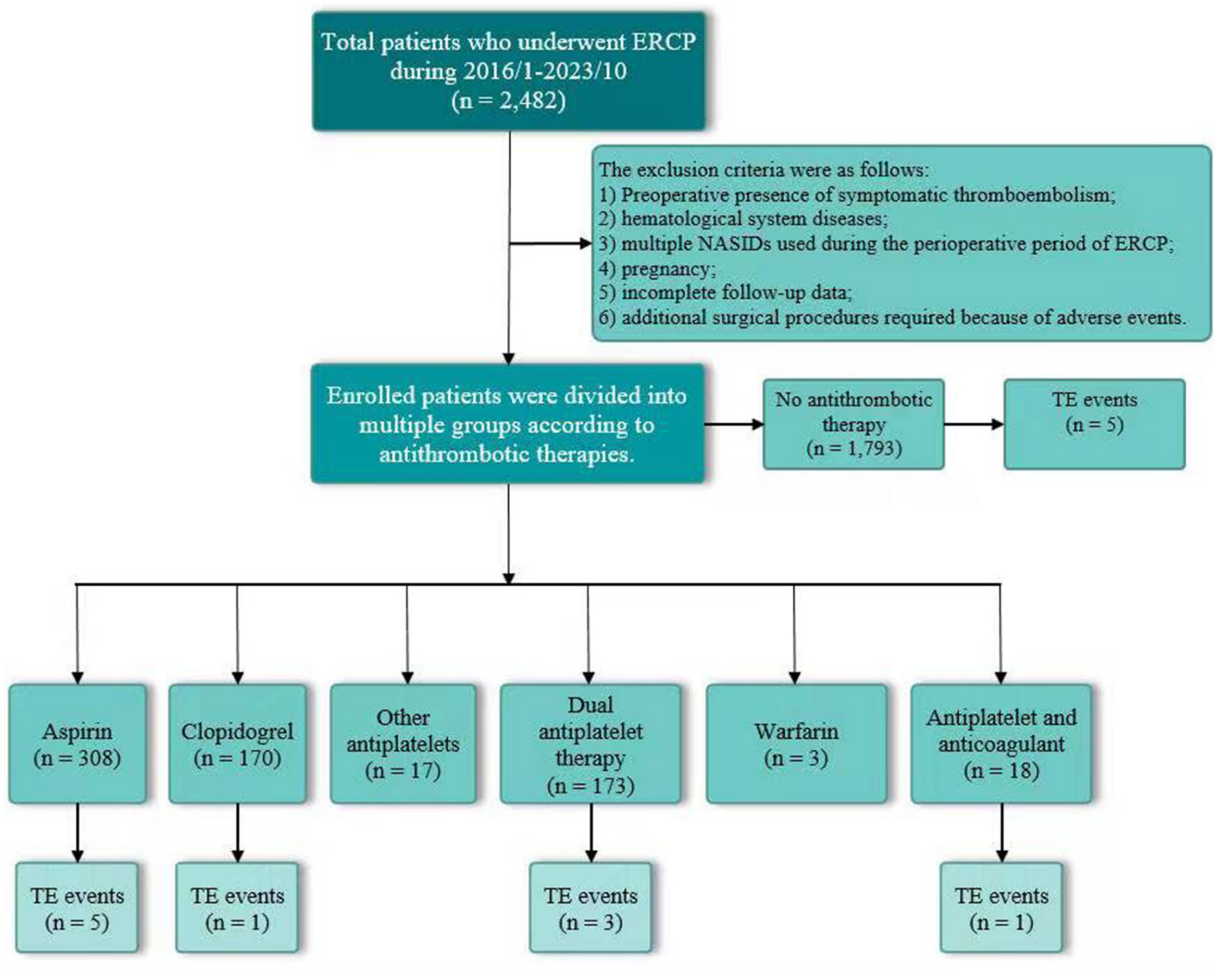
Interruption of antithrombotic therapies and symptomatic thromboembolic events in this cohort. ERCP, endoscopic retrograde cholangiopancreatography; NSAIDs, non-steroidal anti-inflammatory drugs; TE, thromboembolic.

This study was conducted as a retrospective cohort study involving consecutive subjects who had undergone ERCP between January 2016 and October 2023 (*n* = 2,482). Our conventional clinical practices followed the international guidelines on managing antithrombotic drugs for patients undergoing ERCP (9, 13–16). The indications for ERCP included choledocholithiasis, malignant biliary stenosis, acute obstructive cholangitis, and others (15). Clinical data were collected, including age, sex, patient comorbidities, indications for ERCP, ERCP procedure, use of antithrombotic medications, interruption duration of antithrombotic drugs, ERCP complications, and symptomatic TE events. Usage of antithrombotic drugs, including combination therapy, and days withheld prior to ERCP were recorded prospectively during ERCP. Heparin bridging therapy was not used by the patients with antithrombotic agent interruption. The symptomatic TE events were investigated via electronic medical records (*n* = 2,482) or telephone follow-up with outpatients. Specifically, all patients were informed to visit our outpatient clinic for follow-up 1 month after ERCP upon discharge. These data were investigated through electronic medical records. For those without outpatient follow-up, we conducted investigations through telephone follow-up. A one-month observation after ERCP was regarded as the termination point of the event. The inclusion criteria were as follows: (1) signed informed consent; (2) diagnosis of pancreaticobiliary duct disease; and (3) age ≥ 18 years. The exclusion criteria were as follows: (1) preoperative presence of symptomatic thromboembolism; (2) hematological system diseases; (3) multiple non-steroidal anti-inflammatory drugs (NSAIDs) used during the perioperative period of ERCP; (4) pregnancy; (5) incomplete follow-up data; and (6) additional surgical procedures required because of adverse events (Figure 1). This cohort study was approved by the institutional review board (IRB) of Fuyang People’s Hospital, and written consent for ERCP was provided by all of the patients.

### ERCP

All ERCP procedures were conducted by four endoscopists who each had previously performed them skillfully. Each endoscopist had undergone systematic training and learning and had experience with at least 500 cases of ERCP. The patients were placed in a prone position on a mattress and were sedated by an intravenous injection of diazepam. Procedure details included endoscopic papillary balloon dilatation (EPBD), endoscopic sphincterotomy (EST), endoscopic retrograde pancreatic drainage (ERPD), endoscopic retrograde biliary drainage (ERBD), endoscopic nasobiliary drainage (ENBD), intraoperative and postoperative adverse events, and operation-related death. Indometacin suppositories were used as follows: (1) they were given via transrectal administration 15 min before ERCP (50–100 mg per patient); (2) they were used again when patients felt intolerable abdominal pain after ERCP (such as PEP, Visual Analogue Scale ≥4 points) at a dose of 100 mg/time.

### Clinical outcome

Symptomatic TE events were defined as sudden limb swelling accompanied by pain and changes in skin color and temperature, confirmed by ultrasound examination as thromboembolism; sudden breathing difficulties, chest pain, hemoptysis, or coughing, confirmed by computed tomography pulmonary angiography (CTPA) as thromboembolism; sudden focal neurological deficits, hemiplegia, sensory disorders, aphasia, or ataxia, confirmed by cranial magnetic resonance imaging (MRI) as acute cerebral infarction; and sudden chest pain and chest tightness, confirmed as acute myocardial infarction through electrocardiogram and myocardial enzyme examination. ERCP-related bleeding was defined as postoperative black stool or hematemesis with or without reduced hemoglobin levels, or other interventions such as blood transfusion, endoscopy, angiography intervention, or surgical treatment. ERCP-related perforation was diagnosed if there was a visualization of any extra digestive tract structure during the procedure, or leakage of contrast agent outside the wall of the digestive tract. Post-ERCP pancreatitis was defined as new or existing abdominal pain worsening after 24 h following ERCP; serum amylase or lipase levels increase by ≥3 times the upper limit of normal values; and hospital stay extended by ≥2 days. The interruption of antithrombotic drugs was described as 24 h or more before ERCP because of digestive preparation, acute abdominal pain, acute cholangitis, or others. The interruption duration of antithrombotic drugs was described as an empty stomach duration around ERCP without the use of any antithrombotic agents. The interruption usually lasted from 1 to 3 days before ERCP and 1 to 10 days after ERCP. Resumption of antithrombotic drugs was defined as water intake started after ERCP, along with no hematemesis, black stools, or hemoglobin fall of more than 2 g/day. Antithrombotic therapy was restarted as early as possible after ERCP. We followed the guidelines for high-risk and low-risk conditions for symptomatic TE events with minor modifications (9, 16). High-risk conditions for symptomatic TE events were considered malignancy, <3 months after symptomatic venous TE, ischemic heart disease along with previous coronary stent implantation, atrial fibrillation or valvular heart disease along with previous mural thrombus, and stroke with sequelae (for example, hemiplegia and aphasia). Low-risk conditions for symptomatic TE events were considered ischemic heart disease without previous acute myocardial infarction or coronary stent implantation, stable cerebrovascular disease and peripheral vascular disease, atrial fibrillation without mural thrombus, and >3 months after symptomatic venous TE.

The primary outcome was the rate of any symptomatic TE event within 30 days after ERCP.

### Statistical analysis

Categorical variables were described as numbers and percentages (%). Continuous variables were described as the median with an interquartile range (IQR). Missing baseline details were deemed to be missing at random and were supplemented with substituted values obtained through multiple imputations with chained equations. Pearson’s *χ*^2^ test was performed to compare proportions. Then, 95% confidence intervals (95% CIs) and odds ratios (ORs) were calculated to estimate the differences between the groups. The risk of post-ERCP symptomatic TE events was confirmed by multivariate logistic regression. The covariates included in this model were the subject’s sex, age, smoking status, drinking status, body mass index (BMI), diabetes, hypertension, procedure duration, use of rectal indomethacin suppository ≤100 mg, high-risk conditions for TE according to the specific variables, and indications for ERCP. The risk was then expressed as an adjusted OR (aOR). Subgroup analysis was conducted with the covariates in this model, comparing the risk of symptomatic TE events between those treated with indomethacin suppositories >100 mg and interrupting antithrombotic therapy and those treated with indomethacin suppositories ≤100 mg and not on antithrombotic therapy as control. Statistical analysis was performed with SPSS software (version 20.0). All of the tests were Fisher’s exact or two-sided, with *p*-values <0.05 deemed to indicate statistical significance.

## Results

### Subjects’ characteristics

A total of 2,482 subjects were included (Table 1). The median age of the subjects was 69 years (IQR 55–78), and there were 52.98% women. A total of 689 (27.76%) subjects were treated with antithrombotic agents, including 308 (12.24%) with aspirin, 3 (0.12%) with warfarin, 170 (6.85%) with clopidogrel, and 208 (8.38%) with other agents. The remaining subjects were receiving different combinations of anticoagulants and anti-platelet drugs, including dual anti-platelet therapy, antiplatelets and anticoagulants, and other anti-platelets. None of the patients with interruptions of antithrombotic drugs (*n* = 677) received heparin bridging therapy in this study. Patients (*n* = 12) without interrupting their anti-thrombotic drugs were mainly those taking aspirin. In total, 1,274 patients received indomethacin suppositories ≤100 mg because of concerns about the risk of bleeding. Twelve patients continued the use of antithrombotic drugs, and their details are listed in Tables 1, 2. Among these subjects (*n* = 2,482, Table 2), 1900 (76.55%) used indomethacin suppositories. At baseline, 556 and 133 patients were considered to have low-risk and high-risk conditions for TE events, respectively. The most common indication for ERCP was choledocholithiasis (59.75%). EST and EPBD were performed in 2,382 (95.77%) subjects. The median procedure duration was 54.00 min (IQR 42.00–66.00), with 97.38% ENBD.

**Table 1:** Patient’s baseline characteristics.

Patient’s characteristics	Median (IQR)
Number of patients	2,482
Male	1,167 (47.02%)
Age (year)	69.00 [55.00, 78.00]
BMI (kg/m^2^)	22.32 [19.98, 24.08]
Drinker	1,436 (57.86%)
Smoker	1,011 (40.73%)
Indications for ERCP	
Acute obstructive cholangitis	255 (10.27%)
Choledocholithiasis	1,483 (59.75%)
Malignant Biliary Stenosis	65 (2.62%)
Others	679 (27.36%)
ERCP procedures	
EPBD	2,382 (95.97%)
EST	2,013 (81.10%)
ERBD	188 (7.57%)
ERPD	216 (8.70%)
ENBD	2,417 (97.38%)
Procedure duration (min)	54.00 [42.00, 66.00]
Patient comorbidities	
Diabetes	154 (6.20%)
Hypertension	803 (32.35%)
Valvular heart disease	79 (3.18%)
Atrial fibrillation	109 (4.39%)
Ischemic heart disease	775 (31.22%)
Stroke or TIA	583 (23.49%)
Hyperlipidemia	144 (5.80%)
Antithrombotic medications	
No	1,793 (72.24%)
Aspirin	308 (12.41%)
Clopidogrel	170 (6.85%)
Other anti-platelets	17 (0.68%)
Dual antiplatelet therapy	173 (6.97%)
Warfarin	3 (0.12%)
Antiplatelet and anticoagulant	18 (0.73%)

BMI, body mass index; TIA, transient ischemic attack; ERCP, endoscopic retrograde cholangiopancreatography; EPBD, endoscopic papillary balloon dilatation; EST, endoscopic sphincterotomy; ERPD, endoscopic retrograde pancreatic drainage; ERBD, endoscopic retrograde biliary drainage; ENBD, endoscopic nasobiliary drainage; IQR, interquartile range.

Continuous variables were presented as median and IQR.

Categorical variables were presented as numbers with percentages.

**Table 2:** Incidence rates of symptomatic thromboembolic events in different groups.

Variables	No. of patients	No. of symptomatic TE events (*n* %)			Odds ratio^**^
		Within 7 days	7–30 days	Total	
All subjects	2,482				
No antithrombotic agents	1,793	1 (0.06)	4 (0.22)	5 (0.28)	Reference
Any antithrombotic agents	689	9 (1.31)	1 (0.15)	10 (1.46)	5.26 [1.79–15.46]
Anti-thrombotic medications					
Aspirin	308	4 (1.30)	1 (0.32)	5 (1.62)	5.90 [1.70–20.51]
Clopidogrel	170	1 (0.59)	0 (0.00)	1 (0.59)	2.11 [0.25–18.22]
Other antiplatelets	17	0 (0.00)	0 (0.00)	0 (0.00)	–
Dual antiplatelet therapy	173	3 (1.73)	0 (0.00)	3 (1.73)	6.31 [1.50–26.63]
Warfarin	3	0 (0.00)	0 (0.00)	0 (0.00)	–
Anti-platelet and anticoagulant	18	1 (5.56)	0 (0.00)	1 (5.56)	21.03 [2.33–189.75]
Indometacin suppository use*					
≤100 mg	1,274	10 (0.78)	4 (0.31)	14 (1.09)	3.97 [1.43–11.06]
>100 mg	1,208	0 (0.00)	1 (0.08)	1 (0.08)	0.29 [0.04–2.54]
Indications for ERCP					
Acute obstructive cholangitis	255	1 (0.39)	0 (0.00)	1 (0.39)	Reference
Choledocholithiasis	1,483	5 (0.34)	1 (0.08)	6 (0.42)	1.03 [0.12–8.61]
Malignant biliary stenosis	65	1 (1.54)	2 (3.08)	3 (4.62)	12.29 [1.26–120.18]
Others	679	3 (0.44)	2 (0.29)	5 (0.73)	1.88 [0.22–16.21]
PEP^#^					
No	2,376	9 (0.38)	4 (0.17)	13 (0.55)	Reference
Yes	106	1 (0.94)	1 (0.94)	2 (1.88)	0.28 [0.06–1.28]
General anesthesia					
Yes	33	0 (0.00)	0 (0.00)	0 (0.00)	Reference
No	2,449	10 (0.41)	5 (0.20)	15 (0.61)	0.99 [0.99–0.99]

^#^PEP, post-ERCP pancreatitis; ^*^It was a cumulative dosage around ERCP.

^**^The odds ratio was unadjusted.

### Symptomatic TE events after ERCP

There were a total of 15 (0.60%) symptomatic TE events within 30 days after ERCP, and 10 occurred within 7 days. The individual subject details are shown in Table 2. In the group treated with any antithrombotic agent, 9 (1.31%) patients developed symptomatic TE events within 7 days, while 1 (0.15%) individual developed symptomatic TE events beyond 7 days (Table 2). Among the 1,793 subjects not treated with antithrombotic agents, 5 (0.28%) developed symptomatic TE events, with 1 (0.06%) occurring within 7 days and 4 (0.22%) occurring beyond 7 days of ERCP. The symptomatic TE rate in the subjects treated with any antithrombotic agent was 1.46% with an OR of 5.26 (95% CI 1.79–15.46), compared with those not treated with antithrombotic agents. In this study, the patients treated with antiplatelets and anticoagulants at baseline had the highest risk of post-ERCP symptomatic TE events (5.56%; OR 21.03, 95% CI 2.33–189.75), followed by those treated with dual antiplatelet therapy, aspirin, and clopidogrel. The symptomatic TE rate in the subjects on temporary interruption of antithrombotic agents was 1.48% with an OR of 5.36 (95% CI 1.83–15.74), compared with those not treated with antithrombotic agents (Table 3).

**Table 3:** Interruption of antithrombotic agents and symptomatic thromboembolic events.

	No. of patients	No. of symptomatic TE events (*n* %)			Odds ratio^##^
		Within 7 days	7–30 days	Total	
All subjects	2,482				
No antithrombotic agents	1,793	1 (0.06)	4 (0.22)	5 (0.28)	Reference
Any antithrombotic agents	689	9 (1.31)	1 (0.15)	10 (1.46)	5.26 [1.79–15.46]
Indications for antithrombotic					
High–risk condition*	133	7 (5.26)	1 (0.75)	8 (6.01)	22.88 [7.38–70.99]
Low–risk condition	556	2 (0.36)	0 (0.00)	2 (0.36)	1.29 [0.25–6.67]
Interruption of antithrombotic drugs					
No	12	0 (0.00)	0 (0.00)	0 (0.00)	–
Yes	677	9 (1.33)	1 (0.15)	10 (1.48)	5.36 [1.83–15.74]
Interruption duration of antithrombotic drugs**	677				
≤5 days	471	5 (1.06)	0 (0.00)	5 (1.06)	3.83 [1.11–13.31]
5–10 days	192	4 (2.08)	0 (0.00)	4 (2.08)	7.60 [2.03–28.58]
>10 days	14	0 (0.00)	1 (7.14)	1 (7.14)	27.51 [3.00–252.08]

*High-risk conditions for symptomatic thromboembolic events were described as malignancy, <3 months after symptomatic venous thromboembolism, ischemic heart disease along with previous coronary stent implantation, atrial fibrillation or valvular heart disease along with previous mural thrombus, and stroke with sequelae (for example, hemiplegia and aphasia). Low-risk conditions for symptomatic thromboembolic events were defined as ischemic heart disease without previous acute myocardial infarction or coronary stent implantation, stable cerebrovascular disease and peripheral vascular disease, atrial fibrillation without mural thrombus, or >3 months after symptomatic venous thromboembolic.

**Interruption duration of antithrombotic drugs was described as an empty stomach duration around ERCP without the use of any antithrombotic agents.

Resumption of antithrombotic drugs was defined as water intake started after ERCP, along with no hematemesis, black stools, or hemoglobin fall of more than 2 g/day. Antithrombotic therapy was restarted as early as possible after ERCP.

^##The odds ratio was unadjusted.^

The total symptomatic TE event rates in the subjects with high-risk and low-risk underlying conditions were 6.01 and 0.36%, respectively. High-risk conditions were positively associated with symptomatic TE events in the patients treated with antithrombotic drugs compared with the subjects not treated with antithrombotic agents (OR 22.88, 95% CI 7.38–70.99, *p* = 0.00), while low-risk conditions were not associated with a higher risk of symptomatic TE events (OR 1.29, 95% CI 0.25–6.67, *p* = 1.00) (Table 3). The patients who underwent ERCP for malignant biliary stenosis had the highest risk of symptomatic TE events (4.62%; OR 12.29, 95% CI 1.26–120.18, *p* = 0.02).

We further studied the risk of symptomatic TE events among the subjects in whom the treatment with antithrombotic agents was interrupted around ERCP. Notably, none of the patients (*n* = 12) who continued with any antithrombotic agent developed symptomatic TE events (Table 3). In contrast, withholding antithrombotic drugs was associated with higher rates of symptomatic TE events (OR 5.36, 95% CI 1.83–15.74, *p* = 0.002). Compared with the patients not treated with antithrombotic agents, interruption of antithrombotic agents for ≤5 days (1.06%; OR 3.83, 95% CI 1.11–13.31), 5–10 days (2.08%; OR 7.609, 95% CI 2.03–28.58), and >10 days (7.14%; OR 27.51, 95% CI 3.00–252.08) was associated with a higher risk of TE events.

### ERCP-related bleeding (PEB)

**Figure 2:**
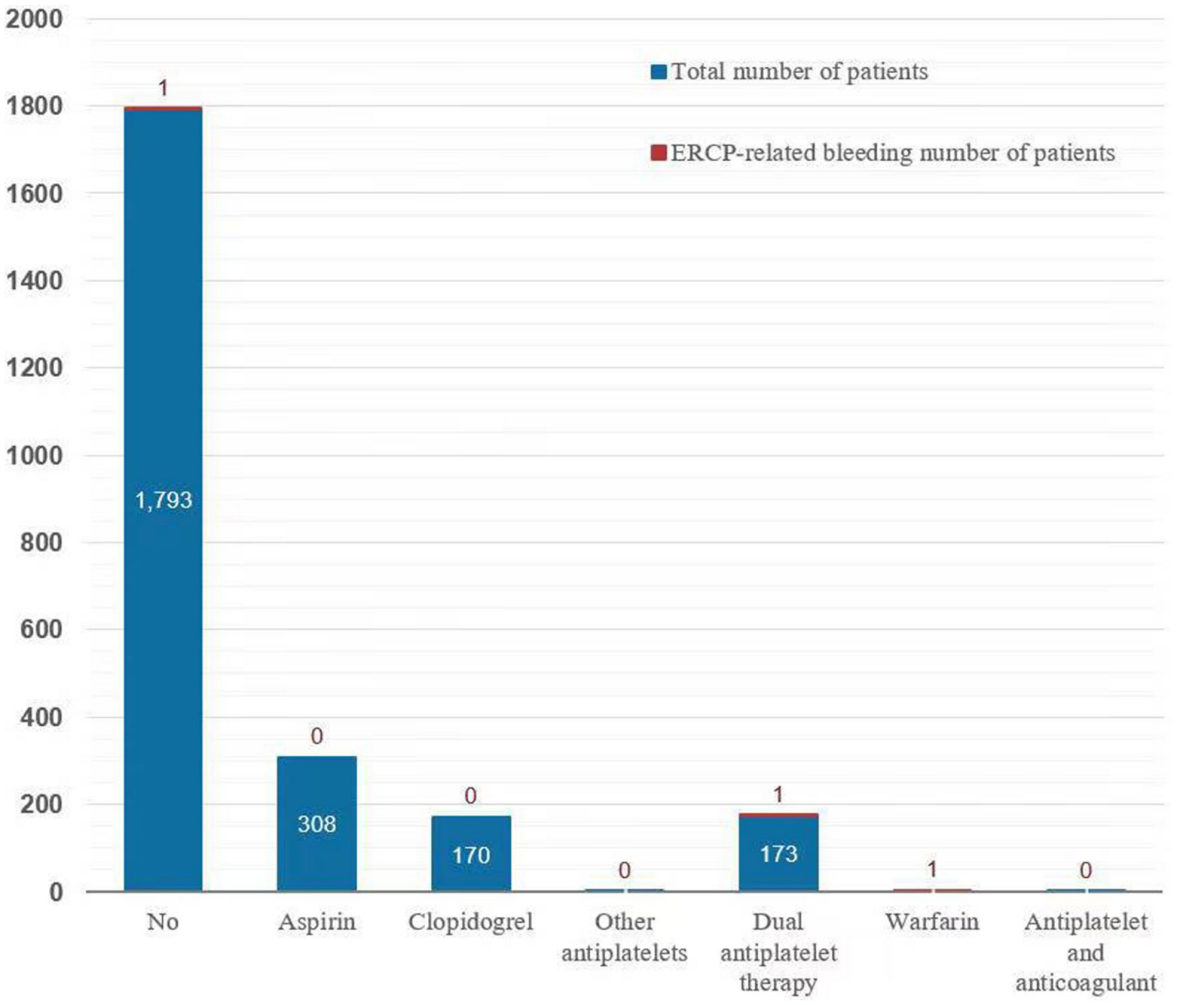
Percentage of ERCP-related bleeding events in patients on various antithrombotic drugs.

Of the 2,482 patients, 1,913 and 469 were predicted to have high and low risk of post-ERCP bleeding based on the EST and EPBD, respectively. Among these subjects, three developed PEB, including 1 patient with indomethacin suppository 0 mg; 1 patient with indomethacin suppository of 100 mg; and 1 patient with indomethacin suppositories of 200 mg. These three patients had comorbidities such as hypertension, ischemic heart disease, or stroke. Two patients (0.08%) on antithrombotic drugs developed PEB, while one (0.04%) patient who was not on antithrombotic drugs developed PEB (OR 0.19, 95% CI 0.02–2.12, *p* = 0.18) (Figure 2). All cases of PEB or perforation were successfully managed by conservative treatment. There were no patients with bleeding-related mortality, and no differences were observed between the groups in terms of blood loss amount or incidence of bleeding. The symptomatic TE event rates in the subjects with high risk and low risk of post-ERCP bleeding were 13 (0.68%, 13/1,913) and 1 (0.21%, 1/469), respectively, which were not different from those in the subjects without EST or EPBD (1.00%, 1/100). During the 1-month follow-up after ERCP, no patients required blood transfusion treatment because of ERCP-related bleeding.

### Symptomatic TE event–associated mortality after ERCP

Two (0.08%) patients died with symptomatic TE events after ERCP, and both patients were on antithrombotic drugs at baseline (one on anti-platelet and anticoagulant, and one on dual anti-platelet therapy). The causes of death in both patients was acute myocardial infarction. These patients had ischemic heart disease with previous coronary stent implantation.

### Risk factors for symptomatic TE events after ERCP

Our multivariate regression analysis with procedure duration, BMI, and age as covariates showed that patients with rectal indomethacin suppositories ≤100 mg and high-risk conditions were at a significantly higher risk of post-ERCP symptomatic TE event. The adjusted OR for rectal indomethacin suppository ≤100 mg was 8.63 (95% CI 1.05–71.15), while the adjusted OR for high-risk conditions was 11.73 (95% CI 2.23–61.70) (Table 4). Thus, patients with high-risk conditions should be closely monitored for symptomatic TE events following ERCP.

**Table 4:** Multivariate regression analyses of risk factors for post-ERCP symptomatic thromboembolic events.

	Adjusted OR [95% CI]	*P*-value
Sex (male)	1.31 [0.14–12.50]	0.81
Age	1.04 [0.99–1.09]	0.09
Smoker	2.81 [0.39–20.34]	0.30
Drinker	0.53 [0.10–2.81]	0.46
BMI	1.21 [0.94–1.57]	0.14
High-risk condition*	11.73 [2.23–61.70]	0.00^#^
Procedure duration	1.01 [0.99–1.04]	0.24
Indomethacin suppository (≤100 mg)	8.63 [1.05–71.15]	0.04^#^
Diabetes	2.78 [0.77–10.05]	0.12
Hypertension	3.29 [0.71–15.19]	0.13

^#^Adjusted ORs, adjusted for sex (male), age, BMI, smoker, drinker, a high-risk condition for TE, any antithrombotic agent used, indications for ERCP (acute obstructive cholangitis, choledocholithiasis, malignant biliary stenosis, and others), procedure duration, diabetes, hypertension, and rectal indomethacin suppository.

^*^High-risk conditions for symptomatic TE events were described as malignancy, <3 months after symptomatic venous thromboembolism, ischemic heart disease along with previous coronary stent implantation, atrial fibrillation or valvular heart disease along with previous mural thrombus, and stroke with sequelae (such as hemiplegia and aphasia).

## Discussion

In the setting of post-ERCP TE events, few studies have evaluated the risk of interrupting antithrombotic agents. The interruption of antithrombotic agents to reduce gastrointestinal bleeding is weighed against the increased risk of TE events (7). For example, Kusunoki’s study showed that the overall frequency of asymptomatic deep vein thrombosis was 10.0% in patients undergoing endoscopic submucosal dissection (ESD) (17). The baseline (*n* = 2,482, Table 1) 30-day post-ERCP symptomatic TE events occurred in 0.60% of the cases in this study. Among the subjects who were using any of antithrombotic agents, there was a higher risk of symptomatic TE events. More importantly, the interruption of any antithrombotic agent was associated with an increased risk of post-ERCP symptomatic TE events. High-risk conditions with antithrombotic indications were positively associated with symptomatic TE events, compared with subjects not treated with antithrombotic agents. Multivariate regression analysis indicated that the subjects with rectal indomethacin suppository ≤100 mg and high-risk conditions had a significantly higher risk of post-ERCP symptomatic TE events. While mortality caused by direct ERCP-associated complications was rare, post-ERCP symptomatic TE events were associated with a mortality rate of 13.3% (2/15), with both deceased patients being on antithrombotic drugs at baseline.

We next determined the underlying bleeding risk of the endoscopic procedure. High-risk endoscopic procedures are associated with potential bleeding. The high-risk procedures include ERCP with sphincterotomy, ESD, or others (14, 18). The low-risk endoscopic procedures include ERCP without sphincterotomy, biliary stent placement, endoscopic papillary small balloon dilation, or others (18). ERCP with sphincterotomy is a procedure with a high bleeding risk (30-day risk of major bleeding >2.0%) based on empiric endoscopic procedural bleeding risk stratification (9). The ASGE and ESGE-BSG guidelines do not suggest interruption of anticoagulants or antiplatelet agents for patients undergoing low-risk endoscopic procedures (14). Only a few studies have reported the risk of hemorrhage with the continuation of antiplatelet agents in patients undergoing ERCP and sphincterotomies. Based on a nationwide database in Japan, EPBD and EST can be safely conducted in patients receiving antiplatelet agents, while users of anticoagulants are at a high risk of hemorrhage (19). Meunier et al. (20) have revealed that patients on oral antiplatelet drugs do not present an increased risk for post–endoscopic papillectomy delayed bleeding. The decision for discontinuation of antithrombotic therapy in the setting of potential gastrointestinal bleeding must be made on an individual basis, based upon gastrointestinal risk and cardiac risk assessments, to discern potential thrombotic and bleeding complications (21). The Asian Pacific Society for Digestive Endoscopy and the Asian Pacific Association of Gastroenterology recommend the interruption of all antithrombotic agents for ultrahigh-risk procedures (10). The American Society of Gastrointestinal Endoscopy practice guideline encourages endoscopists to consider cardiovascular risks and to defer elective procedures in patients in whom a high risk is present (21). Antithrombotic therapies may be continued as mandated by cardiovascular risk (21). It is important to emphasize that the guideline does not mandate interruption of aspirin or NSAIDs for most endoscopic procedures because of the lack of clear evidence that bleeding rates following an endoscopic procedure are adversely influenced by these drugs. For individuals with potential bleeding and high TE risk conditions, such as a recently placed stent, atrial fibrillation or valvular heart disease along with previous mural thrombus, stroke with sequelae, malignancy, or others, individualized risk stratification should be paramount. For patients with antithrombotic therapies, the risk of TE events can be estimated using the CHA2DS2-VASc score, the timing of venous thromboembolism, the type of mechanical heart valves, thrombophilia syndromes, or others (14). For patients taking antithrombotic drugs who require endoscopy, one should consider the following factors: (1) the bleeding risk of the procedure; (2) the urgency of the procedure; (3) the effect of the antithrombotic medications on the bleeding risk; and (4) the risk of a TE event related to periprocedural discontinuation of antithrombotic agents (15).

The probability of a TE event related to the temporary discontinuation of antithrombotic therapy for an endoscopic procedure depends on individual patient characteristics and the indication for antithrombotic therapy (18). In this study, patients treated with antiplatelets and anticoagulants at baseline showed the highest risk of post-ERCP symptomatic TE events (5.56%; OR 21.03, 95% CI 2.33–189.75), followed by those treated with dual antiplatelet therapy, aspirin, and clopidogrel. Endoscopists should be aware of specific clinical risk factors, such as acute myocardial infarction, prior history of stent occlusion, multivessel percutaneous coronary intervention, diabetes, diffuse coronary artery disease, previous venous thromboembolism, stroke or TIA, atrial fibrillation, and valvular heart disease. There is a recommendation that antithrombotic therapy should be resumed upon completion of the procedure. The study by Paik et al. (22) showed that there was no statistically significant difference in post-ERCP delayed bleeding based on the resuming time of the anticoagulant. The benefits of reinitiation of antithrombotic therapy for the prevention of TE events should be weighed against the risk of bleeding associated with the specific agent, the medication time, and procedure-specific circumstances (21). Assessing TE risk stratification before interrupting antithrombotic agents using some known risk assessment tools (such as the CHA2DS2-VASc score and HAS-BLED score) can help clinicians quantify the risk of thromboembolism and bleeding, thereby better guiding anticoagulant therapy decisions. Long-term (>48 h) cessation of anticoagulant therapy is related to TE events, so early resumption within 48 h is proposed in patients at risk of TE events (23). The conventional guidelines recommended reducing the hemorrhage risk that accompanies gastrointestinal endoscopy, but the current guidelines prioritize the reduction of TE risk during the discontinuation of antithrombotic drugs (24). When the advantages outweigh the disadvantages, the guidelines recommend conducting high-bleeding-risk procedures without the interruption of selected antithrombotic drugs (24).

The following guidelines target individuals in elective (planned) endoscopy procedures (9, 14). For patients on warfarin, the guidelines propose continuation as opposed to temporary discontinuation (1–7 days), but if warfarin is held for procedures with high risk of gastrointestinal bleeding, they recommend against bridging anticoagulation unless the patient has a mechanical heart valve. Recent studies have shown that heparin bridging therapy does not reduce perioperative arterial thromboembolism, with a remarkable risk of delayed bleeding (13). For patients on direct oral anticoagulants (DOACs), the guidelines propose temporarily interruption rather than continuation. DOACs are resumed within 2–3 days after high-risk endoscopic procedures once bleeding has been secured. For patients on dual antiplatelet therapy for secondary prevention, the guidelines propose temporary discontinuation of the P2Y12 receptor inhibitor while continuing acetylsalicylic acid. If a patient is on cardiac acetylsalicylic acid monotherapy for secondary prevention, the guidelines recommend against its discontinuation. We reviewed some guidelines to guide the optimal timing of stopping or continuing antithrombotic drugs before and after endoscopy. The current guidelines do not propose stopping antithrombotic drugs for low-risk procedures such as ERCP with stent placement and papillary balloon dilatation without sphincterotomy. However, the guidelines do recommend stopping these drugs for high-risk procedures, and these strategies are individualized by the type of drugs and the risk of procedures. Also, many complicating factors, such as fear of immediate or delayed bleeding, may prevent the consistent application of the guidelines in actual clinical practice (25). Heparin bridging therapy can be chosen for high TE risk when antithrombotic drugs are interrupted. However, heparin bridging therapy increases bleeding risk, with a 20% incidence in bridged patients compared with 1.4% in non-bridged patients (26). In the study by Tomida et al. (13), there was no EST bleeding with heparin replacement. Another study showed that heparin replacement therapy could increase the risk of postoperative bleeding after endoscopic submucosal dissection for early gastric cancer (27). For these conditions, instead of heparin bridging therapy, uninterrupted warfarin or a temporary short discontinuation of DOACs without heparin bridging therapy may be recommended (27). The length of the temporary discontinuation combines with the drugs’ half-life. Short-term discontinuation of antithrombotic agents is safe.

Some underlying conditions may increase the risk of TE events (28), independently of antithrombotic therapy. Malignant tumors lead to TE events through multiple mechanisms, including hypercoagulability, changes in hemodynamics, endothelial damage, and consumption of anticoagulant factors. Atrial fibrillation leads to TE events by altering the hemodynamic state of the atrium, promoting blood clotting tendency, and increasing the susceptibility of specific structures such as the left atrial appendage. Infection leads to TE events through various mechanisms, such as activating inflammatory mechanisms, promoting hypercoagulability, altering blood flow dynamics, and causing tissue damage, etc.

In this study, d-dimer levels did not differ preoperatively between the patients with TE events and others. This may reveal that resuming antithrombotic therapy on time is crucial for preventing TE events. Notably, 0.12% of patients developed PEB. Although continuing indomethacin suppositories may increase the risk of PEB, we found no PEB risk difference between indomethacin suppository use ≤100 mg and >100 mg. Indomethacin can inhibit platelet aggregation and prolong bleeding duration. Its curative effects on platelets disappear within 24 h after drug discontinuation (29). Thus, rectal indomethacin suppositories used in a dose of >100 mg around ERCP may have a lower risk of symptomatic TE events without increasing the risk of bleeding. This may require prospective, multicenter, randomized controlled studies to validate.

Our study provided important real-world clinical data on the rates of post-ERCP symptomatic TE events associated with the interruption of any antithrombotic agent or not. First, a large sample size of 2,482 patients was considered, thereby increasing the reliability of the study as the overall incidence of symptomatic TE events after ERCP was generally low. Second, in patients with symptomatic TE events, we also assessed details regarding the interruption duration of antithrombotic agents, the date of resumption of antithrombotic agents, and reasons for TE-related death. This study had some limitations. First, our results were based on a retrospective study in a single-center endoscopy unit. Second, it is difficult to determine whether the symptomatic TE events were directly related to the ERCP procedure, interruption of any antithrombotic therapy, or underlying diseases. However, a 30-day cutoff is usually used to consider any underlying ERCP-related complications, which may overestimate the risk.

## Conclusion

In this study, the interruption of antithrombotic drugs was found to be associated with higher post-ERCP symptomatic TE events, particularly in high-risk conditions for TE events. Our study indicated that despite the low rate of post-ERCP symptomatic TE events, the risk was increased in individuals on antithrombotic agent therapy. Accordingly, when to stop and resume antithrombotic drugs should be determined based on individual circumstances. Thus, it is important to balance the risk of symptomatic TE events versus bleeding in high-TE-risk patients.

## Data Availability

The original contributions presented in the study are included in the article/supplementary material, further inquiries can be directed to the corresponding author.
